# Managed Care after Acute Myocardial Infarction (MC-AMI) Reduces Total Mortality in 12-Month Follow-Up—Results from a Poland’s National Health Fund Program of Comprehensive Post-MI Care—A Population-Wide Analysis

**DOI:** 10.3390/jcm9103178

**Published:** 2020-09-30

**Authors:** Krystian Wita, Andrzej Kułach, Jacek Sikora, Joanna Fluder, Ewa Nowalany-Kozielska, Krzysztof Milewski, Piotr Pączek, Henryk Sobocik, Jacek Olender, Lucjan Szela, Zbigniew Kalarus, Pawel Buszman, Piotr Jankowski, Mariusz Gąsior

**Affiliations:** 1 First Department of Cardiology, School of Medicine in Katowice, Medical University of Silesia, 40-635 Katowice, Poland; welwetek@poczta.onet.pl; 2 Department of Cardiology, School of Health Sciences in Katowice, Medical University of Silesia, Ziolowa 47, 40-635 Katowice, Poland; 3 Department of Cardiology, Silesian Centre for Heart Diseases, Medical University of Silesia, 41-800 Zabrze, Poland; s.sikora@sccs.pl (J.S.); ewakozielska@wp.pl (E.N.-K.); mgasior@sum.edu.pl (M.G.); 4 Third Department of Cardiology, School of Medicine in Katowice, Medical University of Silesia, 40-635 Katowice, Poland; joanna.fluder90@gmail.com; 5 American Heart of Poland, 43-100 Tychy, Poland; info@ahp-ccrd.org; 6 Voivodeship Specialist Hospital no 5, 41-200 Sosnowiec, Poland; p.paczek@gmail.com; 7 Voivodeship Specialist Hospital no 2, 44-330 Jastrzebie Zdroj, Poland; henryksobocik@poczta.onet.pl; 8 Scanmed, 30-693 Kraków, Poland; olenderjacek@op.pl (J.O.); lucjan.szela@scanmed.pl (L.S.); 9 Department of Cardiology, Congenital Heart Diseases and Electrotherapy, Medical University of Silesia in Katowice, 41-800 Zabrze, Poland; karzab@sum.edu.pl; 10 Department of Epidemiology, Medical University of Silesia, 40-752 Katowice, Poland; pbuszman@oka.pl; 11 1st Department of Cardiology, Interventional Electrocardiology and Hypertension, Institute of Cardiology, Jagiellonian University Medical College, 31-008 Kraków, Poland; piotrjankowski@interia.pl; 12 3rd Department of Cardiology, Medical University of Silesia in Katowice, Silesian Centre for Heart Diseases, 41-800 Zabrze, Poland

**Keywords:** myocardial infarction, cardiac rehabilitation, cardiovascular prevention, post-infarction prognosis, mortality

## Abstract

Introduction: Advances in the acute treatment of myocardial infarction (AMI) substantially reduced in-hospital mortality, but the post-discharge prognosis is still unacceptable. The Managed Care in Acute Myocardial Infarction (MC-AMI) is a program of Poland’s National Health Fund that aims at comprehensive post-AMI care to improve long-term prognosis. The aim of the study was to assess the effect of MC-AMI on all-cause mortality in one-year follow-up. Methods: MC-AMI includes acute MI treatment, complex revascularization, cardiac rehabilitation (CR), scheduled one-year outpatient follow-up, and prevention of sudden cardiac death. In this retrospective observational study performed in a province of Silesia, Poland, we analyzed 3893 MC-AMI participants, and compared them to 6946 patients in the control group. After propensity score matching, we compared two groups of 3551 subjects each. To assess the effect of MC-AMI and other variables on mortality, we preformed a Cox regression. Results: MC-AMI was related with mortality reduction by 38% in a 12-month observation period and the effect persisted even after. Multivariable Cox regression analysis revealed MC-AMI participation to be inversely associated with 1-year mortality (HR 0.52, 95%CI 0.42–0.65, *p* < 0.001). Besides that, older age (HR 1.47/10 y), ST-elevation AMI (HR 1.41), heart failure (HR 2.08), diabetes (HR 1.52), and dialysis (HR 2.38) were significantly associated with the primary endpoint. Among MC-AMI components, cardiac rehabilitation (HR 0.34) and strict outpatient care (HR 0.42) are the crucial factors affecting mortality reduction. Conclusions: Participation in MC-AMI reduced 1-year mortality by 38% and the effect persisted after the program had been completed.

## 1. Introduction

Advances in the acute treatment of myocardial infarction (AMI) significantly reduced in-hospital mortality among acute coronary syndrome patients, but the long-term prognosis in MI survivors still remains a challenge.

The analysis of the post-discharge period shows a particularly high risk of complications within the first several months after MI. According to the European Society of Cardiology (ESC) registries, the mortality rate in the first 12 months after MI reaches up to 12% and is highly variable across Europe. National registries in European countries and in the USA report similar numbers [1,2,3]. These results suggest that efforts should focus on a post-MI care and on the secondary prevention of cardiovascular complications [4,5].

Insufficient control of cardiovascular risk factors, poor compliance and adherence to pharmacological treatment, limited access to cardiac rehabilitation (CR) resulting in the lack of balanced physical activity, and scarce access to outpatient cardiology care are the crucial factors that are thought to be responsible for post-discharge mortality [6,7].

Out of interventional therapies, access to complete revascularization and cardiac implantable electric device therapy are the crucial factors in post-MI prognosis. Early and complete revascularization (both staged angioplasty and surgical treatment) has a pivotal role in the post-MI population [8,9]. The low rate of device implantation (implantable cardioverters-defibrillators with or without resynchronization) is responsible for the increased rate of sudden cardiac death (SCD) [10], which is a significant thread particularly in the first year after MI.

Despite ESC recommendations, the real-world data show that the guidelines are not followed strictly and the coordination of all the key components of post-MI care is crucial to achieve the goal.

The Managed Care in Acute Myocardial Infarction (MC-AMI, KOS-zawal) is a program introduced by Poland’s National Health Fund and Ministry of Health focused at improving long-term prognosis in AMI patients, by providing a comprehensive in-hospital and post-discharge care. The program includes interventional treatment in AMI, staged complex revascularization, cardiac rehabilitation, scheduled follow-up visits, and prevention of SCD in eligible subjects [11]. As it has been proved in a single center analysis, MC-AMI is related to a 45% reduction of major adverse cardiovascular events (MACE) as soon as in 3-months [12] and 50% reduction of major adverse cardiovascular and cerebrovascular events (MACCE) in 12-month follow-up [13]. So far, there were no population or multicenter analyses of the effect of MC-AMI on mortality or even composite cardiovascular endpoints.

The aim of the analysis was to assess the relation between participation in MC-AMI and post discharge all-cause mortality in 12-month follow-up.

## 2. Materials and Methods

We present the results of the retrospective analysis performed in the population of Silesia, Poland (4.5 million inhabitants), where MC-AMI was introduced as a part of a nation-wide strategy of post-MI treatment and secondary prevention. We used data from the SILesian CARDiovascular (SILCARD) registry, which is based on the data from the Regional Department of the National Health Fund in Poland. The analyzed dataset included patients diagnosed with MI from 1 October 2017 to 31 December 2018 and was derived from all 16 cardiology centers in the Silesian Voivodeship involved in the treatment of acute coronary syndromes.

During the analyzed period, we identified 10 844 subjects diagnosed with MI. This included 3898 subjects participating in MC-AMI, and 6946 subjects in a control group. The control group consisted of AMI patients who were hospitalized in the same period but did not consent for participation in MC-AMI program. After 1:1 propensity score matching, two groups of 3551 subjects each were compared.

### MC-AMI—Program Description and Definitions

MC-AMI is a Poland’s National Health Fund and Ministry of Health program of comprehensive care for AMI patients. The program has four core modules: I—hospitalization and acute intervention according to ESC guidelines, II—cardiac rehabilitation (Module II), III—implantation of implantable cardioverter defibrilator (ICD) or chronic resynchronization therapy (CRT-D) in eligible subjects, and IV—12 months of scheduled outpatient cardiology care (at least 4 visits over 12 months).

**Figure 1:**
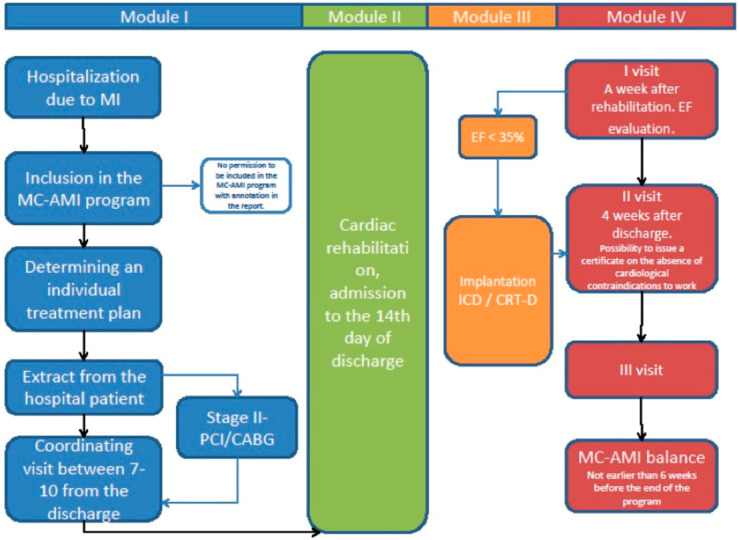
Study flowchart. MC-AMI: Managed Care in Acute Myocardial Infarction, PCI: percutaneous coronary intervention, CABG: coronary artery by-pass grafting, ICD: implantable cardioverter defibrillator, EF: ejection fraction, CRT-D: cardiac resynchronization therapy-defibrillator.

Figure 1 presents the study flowchart. During index hospitalization with interventional treatment of AMI, patients who consented for participation in MC-AMI had a screening visit scheduled 7–10 day after discharge. The visit covered clinical assessment by a cardiology consultant, ECG, and basic blood tests (full blood count, C-reactive protein (CRP), creatinine, sodium, and potassium). Eligible patients with no contraindications were then qualified for cardiac rehabilitation, which was set up to start no later than 14 days after discharge.

Cardiac rehabilitation was performed in a cardiac rehabilitation ward (in-hospital; up to 35 consecutive days) or in an outpatient CR facility (22 days). ECG, echocardiogram, 6-min walk test (6MWT), and treadmill test were performed during CR. Rehabilitation program included supervised physical training, interval training on an ergometer, as well as psychological and educational program, including lifestyle counseling, group therapy, and relaxation sessions.

Upon CR completion, patients were scheduled for outpatient follow-up (Visit 1) 6 weeks after MI-related hospitalization. During visit 1, clinical assessment and echocardiography was performed to identify patients eligible for ICD or CRT implantation. Visit 2 was planned 2–3 months after CR completion or 4 weeks after device implantation. Visit 3 was scheduled upon physician’s discretion, and Visit 4 was performed at the end of 12-month follow-up (FU). The protocol allowed modifying the schedule of MC-AMI program in particular patients based on several factors. The most important were staged revascularization and indication for ICD/CRT-D. Additional visits were scheduled if necessary.

The Ethics Committee of the Medical University of Silesia in Katowice approved the study protocol.

Statistical analysis was performed with SPSS v.25.0 software (IBM Corp, Armonk, NY, USA). Propensity Score Matching (PSM) was performed using pre-specified demographic criteria and clinical variables (Table 1 and Table 2). The data were matched with the Greedy data matching algorithm using Mahalanobis distance within propensity score calipers. Each caliper radius was set to 0.2. Sigma. Propensity score was calculated using logistic regression. Qualitative parameters were compared using Pearson’s chi-square test. Relative risk (RR) ratios with 95% confidence intervals (95% CI) were calculated. To assess the effect of MC-AMI and other clinical variables on the primary endpoint, the Cox proportional hazard model was used. All the variables with *p* < 0.1 in the univariate model were included in the model using backward stepwise Wald’s approach. Estimates of endpoint-free survival from MC-AMI and control were presented using the Kaplan–Meier method and log-rank tests were calculated. A *p* < 0.05 was regarded as statistically significant.

## 3. Results

We primarily analyzed 3898 subjects in the MC-AMI group, and 6946 patients in the control group (Table 1).

After propensity score matching, we selected a group of 7102 well-balanced pairs (3551 in each group) with a good bias reduction in all of the parameters listed in Table 2. The average FU time was 22.2 ± 5.3 months in MC-AMI group and 21.3 ± 6.2 months in control.

### 3.1. Primary End-Point

**Figure 2:**
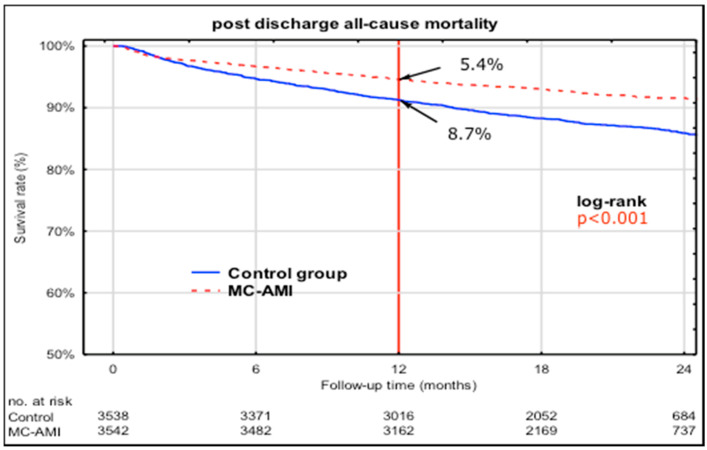
Kaplan–Meier curves showing freedom from all cause mortality—propensity score matching—12-month follow up analysis for the entire study group (7102 subjects). Follow-up extended to 24 months showing persistent effect after the program had been completed. MC-AMI: managed care after acute myocardial infarction.

In a 12-month follow-up, the all-cause mortality rate in the control group was almost twice higher than in the MC-AMI group (8.7% vs. 5.4%. *p* < 0.001), as shown on Kaplan–Meier curves (Figure 2). The positive effect of MC-AMI on all-cause mortality persisted up to 24 months after index AMI and at least a year after participants had completed the program.

Multivariable Cox regression (Table 3) analysis revealed MC-AMI participation to be inversely associated with one-year mortality (HR 0.52. 95% CI 0.42–0.65. *p* < 0.001).

Besides older age, presentation with STEMI, heart failure, diabetes, stroke, chronic obstructive pulmonary disease, malignancy, stroke, and dialysis were significantly associated with the primary endpoint.

### 3.2. Components of MC-AMI

In the MC-AMI group, twice more patients were enrolled in an early in-hospital cardiac rehabilitation program (ECR) (1921 (54%) vs. 971 (27%); *p* < 0.01). Besides that, 1278 (36%) MC-AMI patients participated in ambulatory CR, while this type rehabilitation was only episodically reported in the control group (46 patients, 1.3%).

Implementation of other MC-AMI components in the studied group and respective interventions in the control group are shown in Table 4.

To assess the effect of implementation of particular therapeutic modules on all-cause mortality, we performed a multivariable Cox regression analysis. The analysis was performed in the entire matched population (Table 5A). To assess properly the effect of scheduled FU visits and MC-AMI completion on mortality, we performed the subgroup analysis of patients who survived at least 12 months (effect of variables on 24-month mortality. Table 5B).

## 4. Discussion

The importance of complete revascularization, early cardiac rehabilitation, and strict outpatient care as separate interventions in the post-MI population has been addressed thoroughly in the recent years [14,15]. There are, however, no widely introduced multi-module programs consisting of all key aspects of post-MI care and no reports assessing the effect of such programs on hard clinical endpoints. MC-AMI is a first nation-wide, structured, comprehensive, and supervised care system, close to the optimal and guideline-recommended management, which is hardly met in real-world conditions.

Hereby, we present results of a population-wide analysis of MC-AMI from a Silesian Voivodeship—a province of Poland covering an area of 12,330 square km^2^ with a population of nearly 4.5 million people—which, in our opinion, is a representative scale to draw conclusion of the effect of this nation-wide program.

The primary finding of the analysis is a 38% relative risk reduction of all-cause mortality in a 12-month observation period. In previously published reports, MC-AMI was associated with 45–50% MACE and MACCE reduction [4,5], but these single center analyses were underpowered to prove reduction of mortality. To the best of our knowledge, this is the first study to address the effect of complex care strategy on mortality in post-AMI patients.

In our cohort, in-hospital mortality, interventional treatment, and major comorbidities were balanced in both groups. Thus, the effect on mortality is attributable to the management in the post-discharge period, which includes early cardiac rehabilitation, complete revascularization, device therapy (ICD/CRT), and a strict follow-up. In a light of the current guidelines, CR is one of the crucial steps in post-MI care [16]. In a large meta-analysis, Anderson et al. [17] revealed that CR reduces cardiovascular mortality by 22% but does not affect all-cause mortality. In our study, where participation in CR was one of the most important factors, differing study and control groups, we observed an all-cause mortality reduction, although the effect cannot be attributable to CR only. In a multivariable Cox regression model, participation in MC-AMI was one of the strongest negative predictors of mortality (HR 0.52. *p* < 0.001). In the Cox model assessing the effect of particular modules of MC-AMI, it was CR and completed outpatient care course that reduced the risk of all-cause mortality (HR 0.34 and 0.42, respectively). Moreover, staged revascularization did not reach statistical significance in the Cox model and the ICD implantation increased the HR of death. The latter is obviously not a consequence of a procedure itself, but the effect of a specific subgroup of patients who qualify for ICD (heart failure). A sub-analysis of low-EF group would be necessary to assess the effect attributable to ICD placement, but even in such a cohort the group with ICD is still small.

Thus, in this analysis, CR and outpatient care seem to be the crucial components that affect mortality. Except for single center reports, there is no data in literature to refer our results to. We can, however, compare it to studies assessing the effects of CR. In a CROS meta-analysis, mortality reduction for post-ACS CR participants was 0.49–0.84 in retrospective studies and 0.20–0.69 for prospective ones [18]. In our study, the benefit from MC-AMI seems higher than in most retrospective studies in the CROS meta-analysis. In our opinion, it is the complex approach in MC-AMI that warrants better adverse events reduction over a shorter time. In a large meta-analysis, Anderson et al. [17] revealed that CR reduces cardiovascular mortality by 22% but does not affect all-cause mortality. Our study showed almost 40% reduction of all-cause mortality, which was observed after 12 months, and the trend persisted after the program had been completed. The effect of different aspects of CR and lifestyle modifications in post-MI patients were addressed by a large meta-analysis by Kabboul et al. [19] (50,965 patients, 148 studies). The authors showed that different components of a CR program have a different effect on adverse events reduction. Not only physical training, but also psychosocial management and patient education occurred to influence the endpoints. In our study, the above-mentioned components were included in the CR and FU visits. This may have been responsible for better adherence both with regard to therapeutic lifestyle changes and medical therapy, but the construction of the study does not allow assessing the compliance in MC-AMI group vs. control.

Implantation of cardiac implantable electric devices (CIED) is another key component of MC-AMI. The European Society of Cardiology Guidelines on heart failure management recommend ICD (or CRT-D if eligible) implantation in post-MI patients in whom EF reassessed 6–12 weeks after index MI remains reduced despite optimal medical treatment [20]. The result of the real world registries, however, show that only a fraction of eligible patients is offered a device therapy. The report by Pokorney et al. showed that only 2/3 of the post-MI population with reduced EF at baseline have echo reassessment within 12 months, and even in persistently reduced EF, the device treatment is offered to only 11% of eligible patients [21]. Our results confirm that obligatory EF assessment triples the number of patients referred to ICD/CRT implantation (MC-AMI: 4.3% vs. control 1.4%. *p* < 0.001).

Although the components of MC-AMI may seem nothing more than regular care, the novelty is the approach to execute the guideline-recommended therapeutic interventions, which are normally available within most healthcare systems, but hardly followed accurately. Our results suggest that such an approach, particularly focused on CR and strict follow-up, may warrant better outcome within the same healthcare resources.

### Limitations

The observation is a retrospective analysis with data derived from the registry and National Health Fund database, where some data (e.g., important comorbidities) may have been underreported. Moreover, data on some interesting details (medication on follow up, CR duration and course, and wider range of clinical details including cardiovascular risk factors characteristics) are impossible to gather.

Moreover, as a retrospective analysis, it provides statistical association rather than causal relationships between intervention and the clinical effect.

## 5. Conclusions

Participation in MC-AMI improves prognosis at least by increasing the rate of patients undergoing cardiac rehabilitation, revascularization procedures, and strict post-discharge follow-up. Participation in MC-AMI showed a reduction in 1-year mortality by 38%. The effect was primarily attributable to CR and outpatient care and persisted after the program had been completed.

## Figures and Tables

**Table 1:** Baseline characteristics in unmatched study groups (*n* = 10,844).

Variable	Control Group Standard Care (*n* = 6946)		Study Group MC-AMI (*n* = 3898)		*p*
Age (years); mean ± SD	69.8 ± 11.8		65.1 ± 10.6		<0.001
Female sex	2784	40.1%	1234	31.7%	<0.001
STEMI presentation	1950	28.1%	1641	42.1%	<0.001
Coroary angiography during MI hospitalization	6010	86.5%	3880	99.5%	<0.001
PCI during MI hospitalization	4394	63.3%	3498	89.7%	<0.001
Cardiac rehabilitation	1613	23.2%	2080	53.4%	<0.001
Congestive Heart Failure	1709	24.6%	471	12.1%	<0.001
Arterial hypertension	4992	71.9%	2665	68.4%	<0.001
Diabetes mellitus	2303	33.2%	1134	29.1%	<0.001
Atrial fibrillation	896	12.9%	293	7.5%	<0.001
CKD	448	6.5%	167	4.3%	<0.001
Malignancy	1618	23.3%	793	20.3%	0.004
Stroke	419	6.0%	140	3.6%	<0.001
Scheduled PCI	1095	15.8%	591	15.2%	0.422
Scheduled CABG	95	1.4%	50	1.3%	0.777

MI: myocardial infarction, PCI: percutaneous coronary intervention, CABG: coronary artery bypass grafting, CKD: chronic kidney disease, STEMI: ST-elevation myocardial infarction, SD: standard deviation, MC-AMI: Managed Care in Acute Myocardial Infarction.

**Table 2:** Characteristics of two study groups after propensity score matching; all variables listed matched (*p* = NS).

Variable	Control Group Standard Care (*n* = 3551)		Study Group MC-AMI (*n* = 3551)	
Age (years); mean ± SD	66.1 ± 11.4		66.2 ± 10.1	
Female sex	1179	33.2%	1179	33.2%
STEMI presentation	1384	39.0%	1392	39.2%
Coroary angiography during MI hospitalization	3541	99.7%	3540	99.7%
PCI during MI hospitalization	3124	88.0%	3158	88.9%
Congestive Heart Failure	428	12.1%	466	13.1%
Arterial hypertension	2433	68.5%	2456	69.2%
Diabetes mellitus	1060	29.9%	1053	29.7%
Atrial fibrillation	286	8.1%	289	8.1%
CKD	178	5.0%	161	4.5%
Neoplasms	751	21.2%	747	21.0%
Stroke	136	3.8%	138	3.9%
History of PCI	540	15.2%	552	15.5%
History of CABG	44	1.2%	47	1.3%

CAD: coronary artery disease, MI: myocardial infarction, PCI: percutaneus coronary intervention, CABG: coronary artery bypass grafting, CKD: chronic kidney disease, STEMI: ST-elevation myocardial infarction, SD: standard deviation.

**Table 3:** Cox proportional hazards model—mortality in 12-month observation—matched population—variables included in the final model.

Variable	Hazard Ratio (HR)	95% CI Lower	95% CI Upper	*p* Value
Age (per 10 y)	1.47	1.37	1.57	<0.001
MC-AMI	0.52	0.42	0.65	<0.001
STEMI (vs. NSTEMI)	1.41	1.20	1.66	<0.001
Congestive Heart Failure	2.08	1.78	2.42	<0.001
Diabetes	1.52	1.31	1.76	<0.001
COPD	1.32	1.08	1.62	0.007
Malignancy	1.33	1.14	1.55	<0.001
Stroke	1.60	1.28	2.01	<0.001
Dialysis	2.38	1.60	3.54	<0.001

STEMI: ST-elevation myocardial infarction, NSTEMI: non ST-elevation myocardial infarction, COPD: chronic obstructive pulmonary disease. MC-AMI: managed care after acute myocardial infarction.

**Table 4:** Implementation of the key components of the program in the managed care in acute myocardial infarction (MC-AMI) group and respective interventions in control (if applicable).

Variable	MC-AMI *n* (*n*%)	Control Group *n* (*n*%)	*p* Value
Staged PCI (within index hospitalization)	866 (22.2%)	248 (7%)	*p* < 0.001
Early CABG (index hospitalization or <6 weeks)	259 (7.3%)	76 (2.1%)	*p* < 0.001
Cardiac rehabilitation—in hospital	1921 (54.1%)	971(27%)	*p* < 0.001
Cardiac rehabilitation—outpatient	1278 (36%)	46 (1.3%)	*p* < 0.001
ICD implantation	116 (3.2%)	32 (0.9%)	*p* < 0.001
CRT implantation	41 (1.1%)	19 (0.5%)	*p* = 0.004
MC-AMI 12month follow up completed	2613 (73.6%)	*n*/a	–
Follow up: 2 or less visits completed	923	*n*/a	–
Follow up: 3 visits completed	2060 (58%)	*n*/a	–
Follow up: 4 or more visits completed	568 (16%)	*n*/a	–
Mean number of FU visits (mean. SD)	2.72 ± 1.45	1.2 ± 1.1	*p* < 0.001

PCI: percutaneus coronary intervention, CABG: coronary artery bypass grafting, ICD: implantable cardioverter-defibrillator, CRT: chronic resynchronization therapy, FU: follow-up, *n*/a: not applicable, MC-AMI: managed care after acute myocardial infarction.

**Table 5:** Effect of MC-AMI interventions on mortality—Cox proportional hazards model—mortality in 12-month observation in the entire matched population (**A**) and 24-month mortality in the MC-AMI subgroup who completed 12-month follow-up (FU) (**B**).

**A: Multivariate Cox Regression—Entire Matched Cohort—Effect of Variables on 12-Month Mortality**				
**Variable**	**Hazard Ratio (HR)**	**95% CI Lower**	**95% CI Upper**	***p* Value**
Age (per 10 years)	1.608	1.403	1.842	0.00000
Congestive Heart Failure	1.474	1.107	1.962	0.00794
Diabetes	1.499	1.167	1.926	0.00152
Atrial Fibrillation	1.519	1.094	2.109	0.01254
COPD	1.603	1.165	2.205	0.00374
Dialysis	4.289	2.101	8.756	0.00006
ICD implantation	2.009	1.256	3.213	0.00360
CR—in hospital	0.572	0.430	0.761	0.00013
CR—outpatient	0.262	0.167	0.409	0.00000
**B: Multivariate Cox Regression—12-Months Survivors (MC-AMI Completed)—Effect of Variables on 24-Months Mortality**				
**Variable**	**Hazard Ratio (HR)**	**95% CI Lower**	**95% CI Upper**	***p* Value**
Age (per 10 years)	1.873	1.533	2.289	0.00000
Congestive Heart Failure	1.474	1.107	1.962	0.00794
Atrial Fibrillation	2.080	1.393	3.105	0.00034
COPD	1.779	1.121	2.824	0.01456
CKD	1.859	1.062	3.255	0.02996
Dialysis	3.554	1.179	10.719	0.02434
CR	0.343	0.180	0.652	0.00109
MC-AMI FU completed	0.416	0.291	0.594	0.00000

CKD: chronic. kidney disease, CR: cardiac rehabilitation, FU: follow-up, COPD: chronic obstructive pulmonary disease, ICD: implantable cardioverter-defibrillator, MC-AMI: managed care after acute myocardial infarction.
